# Amelioration of autoimmune neuroinflammation by the fusion molecule Fn14·TRAIL

**DOI:** 10.1186/1742-2094-10-36

**Published:** 2013-03-09

**Authors:** Hodaya Prinz-Hadad, Tehila Mizrachi, Michal Irony-Tur-Sinai, Tatyana B Prigozhina, Alexandra Aronin, Talma Brenner, Michal Dranitzki-Elhalel

**Affiliations:** 1 Laboratory of Neuroimmunology, Department of Neurology and Agnes Ginges Center for Human Neurogenetics, Hadassah-Hebrew University Medical Center, POBox 12000, Jerusalem 91120, Israel; 2 Department of Nephrology and Hypertension Services, Hadassah-Hebrew University Medical Center, Jerusalem, Israel; 3 Department of Neurology, Hadassah-Hebrew University Medical Center p.b.x 12000, Jerusalem 91120, Israel

## Abstract

**Background:**

Multiple sclerosis (MS) is a, T cell-mediated autoimmune disease, the management of which remains challenging. The recently described fusion protein, Fn14·TRAIL, combining the extracellular domain of Fn14 (capable of blocking the pro-inflammatory TWEAK ligand) fused to the extracellular domain of the TRAIL ligand (capable of sending apoptotic signals through its receptors on activated inflammatory cells) was designed to modulate the immune system as an anti-inflammatory agent. The present study explores the efficacy of this purified protein as an anti-inflammatory agent, using the animal model of MS - experimental autoimmune encephalomyelitis (EAE).

**Methods:**

EAE was induced by myelin oligodendrocyte glycoprotein (MOG). Fn14·TRAIL or vehicle were injected daily for 4 to 16 days, at different time points after disease induction. Animals were examined daily and evaluated for EAE clinical signs. Lymphocytes were analyzed for *ex vivo* re-stimulation, cytokine secretion, transcription factor expression and subtype cell analysis. Spinal cords were checked for inflammatory foci. The Mann- Whitney rank sum test, Student’s *t*-test or ANOVA were used for statistical analysis.

**Results:**

Significant improvement of EAE in the group treated with Fn14·TRAIL was noted from day 6 of disease onset and lasted until the end of follow-up (day 40 from disease induction), even in animals treated for 4 days only. Clinical improvement was linked to decreased lymphocyte infiltrates in the central nervous system (CNS) and to decreased Th1 and Th17 responses and to increased number of T- regulatory in the treated mice. No liver or kidney toxicity was evident. *In vitro* assays established the ability of Fn14·TRAIL to induce apoptosis of T cell lines expressing TRAIL receptors and TWEAK.

**Conclusions:**

In this study we established the potency of Fn14·TRAIL, a unique fusion protein combining two potentially functional domains, in inhibiting the clinical course of EAE, even when given for a short time, without apparent toxicity. These findings make Fn14·TRAIL a highly promising agent to be used for targeted amelioration of neuro-inflammatory processes, as well as other autoimmune pathologies.

## Introduction

Multiple sclerosis (MS) is an autoimmune disease in which T cells are sensitized against myelin components [1]. Upon entering the central nervous system (CNS), these encephalitogenic T cells are activated by resident antigen-presenting cells and initiate a cascade of inflammatory damage. The initial inflammatory phase is followed by a phase of selective demyelination and, finally neurodegeneration [2,3]. Today, MS management consists of classical anti-inflammatory agents for acute relapses, and immunosuppression or immunomodulatory drugs given as maintenance and aimed to prevent relapses and slowing down of disability progression [4].

Experimental autoimmune encephalomyelitis (EAE) is the animal model used to study human MS and for investigation of neuroinflammation and autoimmunity in general. The inflammatory process involves the extravasation of activated T cells through blood vessel walls and the activation of CNS immunocompetent cells, which accumulate at the inflammatory sites. The initial inflammatory phase is followed by demyelination and neurodegeneration [4]. EAE is widely used for the study of the underlying pathology of MS, and it has also proved effective in the development of new therapies [5]. Effective treatment should target the pathogenic, myelin-specific T cells in conjunction with preventing their trafficking to the target in the CNS.

Tumor necrosis factor-related apoptosis-inducing ligand (TRAIL), has been shown to play an important role in attenuating disease severity in myelin oligodendrocyte glycoprotein (MOG)-induced EAE. TRAIL was found to inhibit the proliferation of encephalitogenic T cells in EAE, while chronic TRAIL blockade by soluble TRAIL receptor was found to exacerbate EAE and enhance MOG-specific Th1 and Th2 responses [6]. When EAE was induced by adoptive transfer of genetically modified embryonic stem cell-derived dendritic cells (ES-DC) presenting MOG and expressing TRAIL, the disease was less severe than when it was induced by transferring ES-DC presenting MOG but not expressing TRAIL [6]. Of note, these adoptively transferred, genetically modified ES-DCs, presenting both MOG and TRAIL, also protected mice from developing EAE induced by myelin basic protein, the protection mediated by Foxp3 regulatory T cells [7].

Another TNF family member that has gained attention for its role in EAE is the TNF-related weak inducer of apoptosis (TWEAK). It is a type II membrane protein containing 249 amino acids. Although widely expressed at the mRNA level [8,9], membrane-anchored TWEAK protein has been found consistently only in interferon-(IFN) γ-activated monocytes [10]. The TWEAK receptor, fibroblast growth factor-inducible-14 (Fn14, TNFRSF12A), is a type I transmembrane protein with 129 amino acids and lacks a cytoplasmic death domain [11,12]. Fn14 protein expression has been described in fibroblasts [11], endothelial [13,14], glioma [15] and neuronal cells [16]. Functionally, TWEAK promotes angiogenesis [14,17], migration of endothelial and glioma cells [12,15] and has pro-inflammatory properties. TWEAK expression has been shown to be upregulated in EAE, and transgenic mice over-expressing soluble TWEAK in the liver displayed a significant decline of the disease [18]. It has been suggested that TWEAK, by acting at the endothelial blood–brain barrier (BBB) or in the CNS, might promote leukocyte migration into the CNS parenchyma, resulting in exacerbation of the disease [13]. Importantly, blocking the interaction between TWEAK and its receptor by neutralizing Abs against either of them ameliorated clinical signs of EAE in mice and rats [19].

Recently, a chimeric protein combining the ability to interfere with both TRAIL signaling and the TWEAK (Fn14 axis, Fn14·TRAIL) was described [20]. Continuous Fn14•TRAIL expression in EAE mice by a transposon-based eukaryotic expression vector decreased disease severity. This was reflected by a decrease in clinical score, disease incidence, and CNS inflammation.

In the present study we tested the efficacy of purified Fn14·TRAIL protein injected subcutaneously (s.c.) in a chronic EAE model induced by MOG_35-55_ in C57 bl/6 mice. Our results show that Fn14·TRAIL ameliorated clinical and pathological parameters of EAE, reducing CNS inflammation and encephalitogenic T-cell reactivity. Treatment affected Th1 and Th17 lineages and reduced the expression of CCR5 + cells.

## Materials and methods

### Protein production

Human Fn14·TRAIL was produced and purified by Cobra Bio-manufacturing (Keele, UK). Chinese hamster ovary (CHO) cells were stably transfected with a DNA construct expressing the human Fn14·TRAIL gene driven by the cytomegalovirus (CMV) promoter. A stable cell clone was isolated and fermented in a serum-free medium. Fn14•TRAIL was purified from the medium, using chromatographic methods, to >95% purity and stored at -80°C. The amino acid sequence of the Fn14•TRAIL protein is as follows: *MRALLARLLLCVLVVSDSKG***EQAPGTAPCSRGSSWSADLDKCM DCASCRARPHSDFCLGCAAA PPAPFRLLW** RG PQ RVAAHITGTR GRSNTLSSPN SKNEKALGRK INSWESSRSG HSFLSNLHLR NGELVIHEKG FYYIYSQTYF RFQEEIKENT KNDKQMVQYI YKYTSYPDPI LLMKSARNSC WSKDAEYGLY SIYQGGIFEL KENDRIFVSV TNEHLIDMDH EASFFGAFLVG. The *ITALIC* sequence represents the signal-peptide of the human urokinase protein, utilized to secrete Fn14·TRAIL from the cell, and is removed from the mature protein. The amino acid sequence of the extracellular domain of human Fn14 (amino-acids 1 to 52 of the mature protein, marked in bold letters) are directly linked to the extracellular domain of human TRAIL (amino-acids 53 to 217 of the mature protein, non-bold letters).

### Animals and disease induction

Female pathogen-free mice (7 to 8-weeks-old) were purchased from Harlan Laboratories, Rehovot, Israel, and housed in the animal facility of the Hebrew University Medical School in accordance with the National Institute of Health guidelines. All experiments were approved by the Animal Care Committee of the Hebrew University.

EAE was induced as previously described please (21) by injecting 0.2 ml of emulsion containing 125 μg of MOG _35–55_ peptide (Sigma, Israel) and 1 mg of heat-killed *Mycobacterium tuberculosis* extract H37RA (Difco, Detroit MI, USA) in complete Freund adjuvant (CFA) oil s.c. into the left flank of C57BL/6 mice. In addition, the mice received 300 ng of pertussis toxin (List Biological Industries, San Diego CA, USA) intraperitoneally (i.p.) on days 0 and 2. An additional injection of MOG _35–55_ peptide in CFA was delivered into the right flank 7 days later. The mice were treated with daily s.c injections of 50 to 200 μg Fn14·TRAIL in a total volume of 200 μL, or vehicle as control, the starting day after EAE induction varying in different experiments. All the animals were examined daily and evaluated for clinical signs of disease.

The first clinical signs appeared on days 10 to 18 post-immunization. The clinical status of the mice was graded as follows: 0, without clinical disease; 1, tail weakness; 2, hind limb weakness sufficient to impair righting; 3, one plagic limb; 4, paraplegia with forelimb weakness; 5, quadriplegia; and 6, death.

### *Ex vivo* restimulation and cytokine excretion assays

Pooled spleen and inguinal and mesenterial lymph node (LN) cells were harvested from MOG_35-55_ immunized mice treated with Fn14·TRAIL or vehicle, at different time points after immunization. For the proliferation assays, 2 × 10^5^ cells were cultured in RPMI 1640 supplemented with fetal calf serum (FCS) 5%, 1 mM sodium pyruvate, nonessential amino acids, 2 mM L-glutamine, 100 units/ml penicillin, 100 μg/ml streptomycin and 10 μM β-mercaptoethanol in 96 round-bottom plates, in a total volume of 0.2 ml. The cultures were stimulated with MOG _35–55_ (100 μg/ml) or Concanavalin A (ConA; 10 μg/ml, Sigma Aldrich St. Louis MO, USA). At 48 h, the cultures were pulsed with 1 μCi/well [^3^H]thymidine (Amersham Pharmacia Biotech, UK) for 18 h. The cells were then harvested with a semi-automated automatic harvester onto a glass fiber filter, and radioactivity was determined by liquid scintillation. For cytokine assays, harvested cells were cultured in the same medium, stimulated with MOG_35-55_ (100 μg/ml) and incubated for 24 to 72 h. The conditioned media were collected, and cytokine concentrations were determined by quantitative ELISA, using paired mAb specific for the corresponding cytokine, according to the manufacturer’s instructions (BD Pharmingen).

#### Flow cytometry

Surface markers of lymphocytes from pooled LN cells were evaluated at various time points after EAE induction. Cells (0.5 × 10^6^ cells/ sample) were immunostained with the appropriate dilutions of the labeled antibodies in the presence of Fc blocker (1:300, BD Pharmingen, USA) and analyzed by FACS scan. The following antibodies were used: PE-conjugated rat anti mouse CD25 (Serotec, UK), anti CD11b (Pharmingen, San Diego CA,USA), anti-CCR5 (eBioscience) and anti rat IgG_2bκ_ (Pharmingen, USA); FITC-conjugated rat anti-mouse CD4 (BioLegend, San diego CA, USA), anti-mouse CD19, B220, CD3 all from eBioscience, USA, and anti rat IgG_2aκ_ from Pharmingen, USA. For determination of the frequency of CD4^+^CD25^+^FOXP3^+^ cells, which were defined Treg cells, a specific mouse regulatory T-cell staining kit (eBioscience, San-Diego CA, USA) was used. The assay was done according to the manufacturer’s instructions.

#### Quantitative real-time PCR

The semiquantitative real-time PCR reaction was performed as described previously [21,22]. The results for gene expression were normalized to the hypoxantine phosphoribosyltransferase (*HPRT*) gene. The primers used were:

1. HPRT forward TCCTCCTCAGACCGCTTTT

2. HPRT reverse, CCTGGTTCATCATCGCTAATC

3. GATA-3 forward: GCAGAAAGCAAAATGTTTGCTTC

4. GATA-3 reverse, GAGTCTGAATGGCTTATTCACAAATG

5. RORγt forward: CCCTGGTTCTCATCAATGC

6. RORγt reverse: TCCAAATTGTATTGCAGATGTTC

7. T-bet forward CAGTTCATTGCAGTGACTGCCTAC

8. T-bet reverse CAAAGTTCTCCCGGAATCCTTT.

#### Western blot

Upon termination of the experiments, blood was drawn from the mice and the serum was collected after centrifugation. Fn14·TRAIL (100 ng), Fn14 (recombinant mouse; ALEXIS Biochemicals, San Diego, CA, USA) or soluble TRAIL (hTRAIL/Apo2L; PeproTech, Rocky Hill, NJ, USA) were diluted with PBS to a total volume of 20 μL and mixed with Laemmli sample buffer (Bio-Rad) at a 1:1 ratio with or without betamercaptoethanol, and heated or not heated for 10 min at 95°C. They were then loaded onto 10% SDS-PAGE. Following electrophoresis, the gels were blotted onto nitrocellulose membranes (Schleicher & Schuell), blocked with 5% milk/PBS, and probed overnight with either anti-TRAIL (1:000; ALEXIS biochemicals, San Diego, CA, USA), anti-Fn14 (1:500; ALEXIS biochemicals) or serum from the different experimental groups or control mice. After extensive washing, the blots, were incubated with HRP-conjugated matching secondary relevant Abs (Bio-Rad, Hercules, CA, USA) or anti-mouse Abs, and developed with enhanced chemiluminescent substrate (Sigma-Aldrich St. Louis MO, USA) before exposure to X-ray film. The films were scanned and quantified by ImageMaster VDS-CL (Amersham Pharmacia Biotech).

#### Pathology

To test whether Fn14·TRAIL has a toxic effect on the liver or other organs, on the day of sacrifice, livers, lungs, kidneys, guts and brains were harvested from mice in the different experimental groups, or control group, and kept in formalin. Hematoxylin-eosin stained slides were prepared, and sent for blind pathological evaluation. For evaluating disease involvement of the spinal cord, tissues were sampled on day 20, corresponding to the inflammatory peak of the disease, before the chronic phase. Anesthetized mice were sacrificed by total body perfusion with cold 4% paraformaldehyde in PBS, pH 7.3. Lumbar spinal cords, where inflammatory foci predominate in our model, were cut into three coronal segments, fixed in 4% paraformaldehyde, dehydrated and embedded in paraffin. Longitudinal sections were cut to include the majority of the length of the spinal cord, containing both gray and white matter and stained with Hematoxylin-Eosin. Inflammatory foci containing at least 20 perivascular mononuclear cells were counted in each section.

#### Apoptosis detection

The Jurkat T cell leukemia cell line was used for this purpose. A total 2 × 10^5^ cells incubated with or without the indicated proteins for 48 h, were collected, and stained with annexin V- fluorescein isothiocyanate (FITC) and propidium iodide (PI), using a MEBCYTO Apoptosis Kit (MBL, Japan) according to the manufacture protocol. Stained cells (2.5 × 10^4^ cells per sample) were counted with a BD FACS Calibur flow cytometer; the results were analyzed using CellQuest software.

#### Pharmacokinetics

C57BL/6 male mice were selected for this experiment. Fn14·TRAIL (100 μg) was administered as a single s.*c.* injection*.* Blood samples were collected at 0, 1, 4, 8, 24 and 48 h after administration. Serum was analyzed by ELISA for total Apo2L/TRAIL concentrations, using a TRAIL/Apo2L ELISA Kit,, DIACLONE, France), according to the manufacturer’s instructions.

### Statistical methods

Group differences were analyzed using Student’s *t*-test for two groups or by one way analysis of variance (One Way ANOVA) for several groups, according to Holm-Sidak. Differences in disease severity were analyzed using the Mann–Whitney rank sum test. *P* <0.05 was considered significant.

## Results

### Fn14·TRAIL inhibits early T cell response to MOG_35-55_ immunization

To assess the influence of Fn14·TRAIL on CNS inflammation, we treated MOG-induced EAE mice from the day of induction with different doses of Fn14•TRAIL or vehicle, immediately after immunization. On day 9 after immunization the mice were sacrificed, and the spleen and draining lymph-nodes were harvested and weighed. Mice treated with Fn14·TRAIL had smaller spleens, and fewer splenocytes. Peripheral blood counts, indicated, however, that this was specific to the spleen as leukopenia or lymphopenia were not found following treatment.

**Figure 1:**
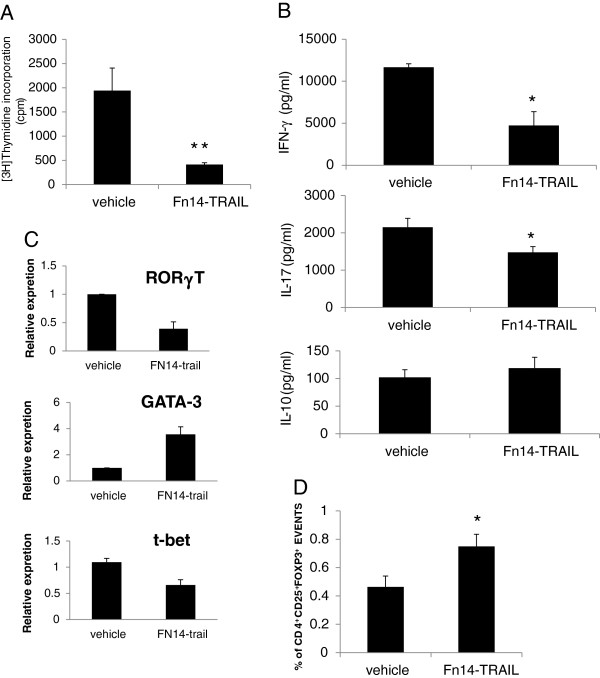
**Reduced T-cell reactivity in mice treated with Fn14·TRAIL during induction of experimental autoimmune encephalomyelitis (EAE).** Mice were treated with 200 μg/day Fn14·TRAIL subcutaneously (s.c.) for 9 days. Pooled lymphocytes were obtained 9 days post-EAE induction. **(A)** Fn14·TRAIL reduced T cell proliferation induced by encephalitogenic Ag MOG_35-55_. **(B)** Fn14·TRAIL reduced production of Th1 lineage cytokine IFN γ and Th17 lineage IL-17 with no significant change in Th2 cytokine IL-10. **(C)** Fn14·TRAIL effect on mRNA levels of Th2 lineage transcription factor GATA-3, Th17 RORγT and T-bet factors. **(D)** Splenocytes collected on day 9 were immune-stained and analyzed by fluorescence-activated cell sorting (FACS) for the percentage of CD4^+^CD25^+^FoxP3^+^ cells. (n = 5 in each group). *P <0.05, **P <0.01.

In addition, pooled splenocytes or regional lymph-nodes lymphocytes from treated and nontreated animals were *ex vivo* re-stimulated with MOG_35-55_. Splenocytes and lymphocytes from mice treated with Fn14·TRAIL proliferated to a lesser extent when stimulated (Figure 1A). Interestingly, pooled lymphocytes from Fn14·TRAIL treated mice responded normally to Concanavalin A stimulation. In opposite, the reactivity of the splenocytes from the Fn14·TRAIL to treated group to Concanavalin A was significantly inhibited to 48% of the activity of vehicle-treated mice. Furthermore, interferon γ (IFN γ) and IL-17 levels in the conditioned media of these cells were significantly reduced (Figure 1B). However, the level of IL-10 did not differ significantly between the groups, as well as IL-4 (not shown). Taken together, these findings suggest that Fn14·TRAIL interferes with the initial T cell response to MOG_35-55_. We tested also the effect of Fn14·TRAIL on the mRNA levels of specific TFs responsible for Th1, Th2 and Th17 lineage differentiation. As can be seen in Figure 1C, Fn14·TRAIL reduced the level of T-bet expression by 50% and of ROR-γt by 3.3 fold, similar to the reduction in the level of IFN γ and IL-17. In addition, an increase of 2.5 fold in the expression of GATA-3 the Th2 TF, despite the fact that the IL-10 and IL-4 levels did not change significantly. Cumulatively, the results support a role for Fn14·TRAIL in skewing Th lineage development from Th1 and Th17 to Th2.

We next tested the Fn14·TRAIL effect on immune cell populations. The phenotype of the splenocytes was characterized by fluorescence-activated cell sorting (FACS) analysis on day 9. There was no significant difference in the percentage of CD3, CD4, CD8, CD25, B220, CD19, CCR5 and CD11b-expressing cells between the placebo-treated and the Fn14·TRAIL-treated mice. Interestingly, there was a significant increase in the percentage of CD4^+^CD25^+^FoxP3^+^ T regulatory cells (Figure 1D).

### Fn14•TRAIL treatment decreases the severity of experimental autoimmune encephalomyelitis

**Figure 2:**
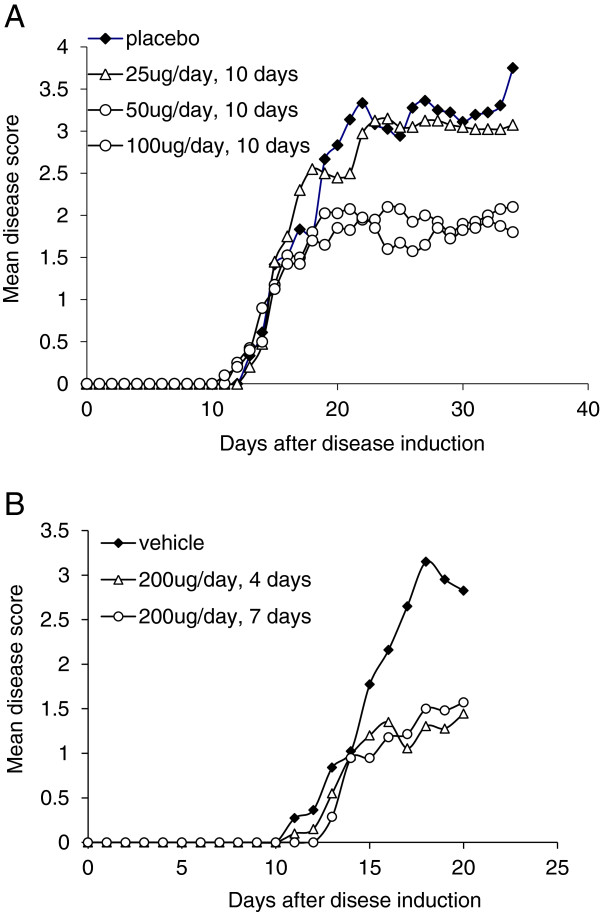
**Treatment with Fn14·TRAIL ameliorated the clinical course of experimental autoimmune encephalomyelitis (EAE). (A)** Daily treatment with Fn14·TRAIL 25–100 μg/day subcutaneously (s.c.) for 14 days from day 10 post-induction. Doses of 50 and 100 μg/day significantly ameliorated the clinical score of EAE (see also Table 1). **(B).** Shorter treatment duration with Fn14·TRAIL 200 μg/day for 7 or 4 days, also ameliorated the clinical score of EAE (see also Table 1).

To evaluate the effect of Fn14•TRAIL *in vivo*, we treated immunized animals with daily s.c. injections of 25 to 100 μg Fn14·TRAIL, starting on the day of immunization, with no significant change in disease severity (data not shown). Interestingly, when treatment was initiated on day 10 after disease induction and lasted for 14 to 16 days, significant abrogation of disease severity was detected in mice treated with 50 to 100 μg of Fn14•TRAIL, (Figure 2A and Table 1). As treatment with up to 100 μg a day was well tolerated by the mice, we next tried to shorten treatment duration, using 200 μg of Fn14·TRAIL per mouse per day, and similar results were obtained. Treatment courses of 7 or even 4 days resulted in a significant and long-lasting effect on disease severity (Figure 2B and Table 1).

**Table 1:** Amelioration of experimental autoimmune encephalomyelitis (EAE) by Fn14·TRAIL

**Treatment**	**Disease mean severity**
Vehicle	2.4 ± 0.2
Fn14·TRAIL 25 μg/day (14-day treatment)	2.3 ± 0.2
Fn14·TRAIL 50 μg/day (14-day treatment)	1.6 ± 0.1*
Fn14·TRAIL 100 μg/day (14-day treatment)	1.5 ± 0.1*
Fn14·TRAIL 200 μg/day (14-day treatment)	1.2 ± 0.1*
Fn14·TRAIL 200 μg/day (7-day treatment)	1.4 ± 0.3*
Fn14·TRAIL 200 μg/day (4-day treatment)	1.2 ± 0.3*

The data are expressed as mean disease score ± SE, in 21 control-vehicle treated mice, and in 10 mice in each treatment group. Treatment started from day 10 post-induction and lasted for the time periods indicated. *P <0.05 versus control.

### Inhibition of central nervous system inflammation by Fn14•TRAIL

**Figure 3:**
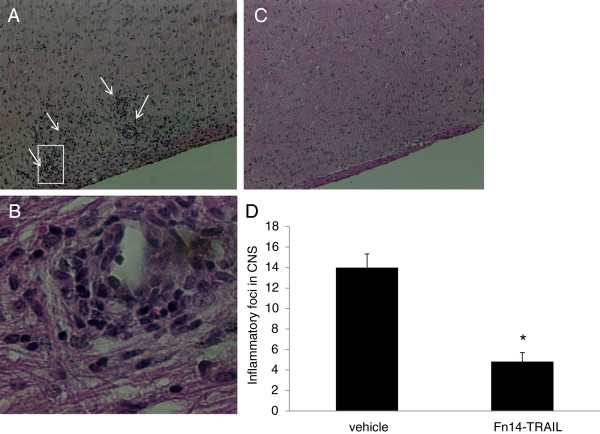
**Treatment with Fn14·TRAIL improves central nervous system (CNS) inflammation and reduces experimental autoimmune encephalomyelitis (EAE) lesions.** Spinal cords of mice from control and Fn14·TRAIL groups were removed at disease peak (day 20 post-induction). Hematoxylin-eosin staining revealed marked perivascular, meningeal and white matter infiltration in EAE mice treated with vehicle (**(A)** 10X magnifications and **(B)** 40X magnification of one perivascular inflammatory lesion). Following treatment with Fn14·TRAIL 200 μg/day for 7 days, a marked and significant reduction in inflammation was observed **(C, D)** *P <0.05.

The significant difference in clinical disease score between Fn14·TRAIL-treated and placebo-treated mice was consistent with the histological analysis of spinal cord tissue removed at the peak of the disease (day 20 post-induction). In the placebo-treated group, pronounced perivascular as well as meningeal and white matter infiltration were observed (Figure 3), whereas much less inflammation was found in the Fn14·TRAIL-treated group. A comparison of the number of inflammatory infiltrates showed a 64% reduction following treatment (Figure 3D)*.*

### Fn14·TRAIL inhibits Th1 and Th17 responses during the course of experimental autoimmune encephalomyelitis

**Figure 4:**
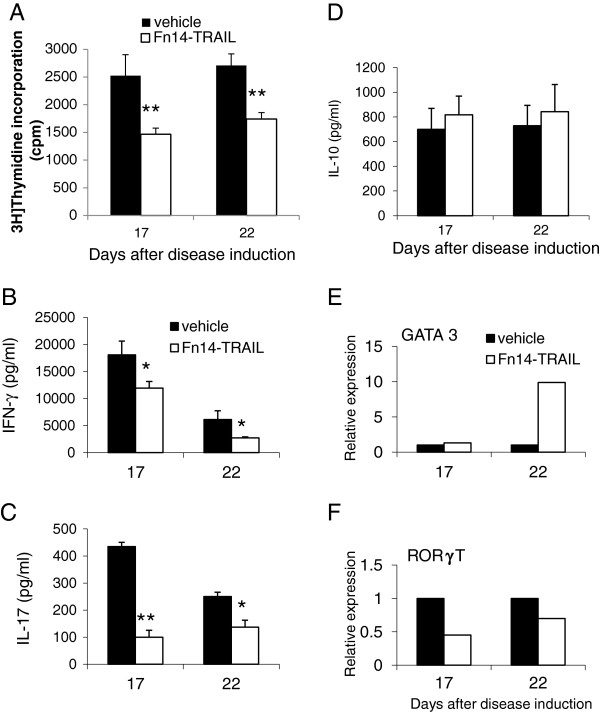
**Treatment with Fn14·TRAIL suppresses T-cell reactivity during various stages of experimental autoimmune encephalomyelitis (EAE) course. (A)** T-cell proliferation in response to encephalitogenic peptide MOG_35-55_ was assessed upon termination of treatment (day 17 post-induction) and 5 days later (day 22). Animals received 200 μg/day for 7 days from day 10 post- EAE induction. **(B – D)** Fn14·TRAIL reduced production of Th1 and Th17 cytokines IFNγ and IL-17, with no change in production of Th2 cytokine IL-10. **( E-F)** Fn14·TRAIL effects were determined on mRNA levels of the Th2 lineage-specific transcription factor (TF) GATA-3, with increased expression (**E**), and a reduction in Th17 TF RORγT (**F**). *P <0.05, **P <0.01

To further corroborate the effects of Fn14•TRAIL treatment on the encephlitogenic T cells, when treatment was initiated on day 10 post-immunization and lasted for 7 days, splenocytes were tested on day 17 upon termination of treatment and 5 days later (day 22). T cell proliferation was reduced by 42 and 36% respectively following Fn14•TRAIL treatment, as was the production of the pro- inflammatory cytokines INFγ (by 34 and 55% respectively) and IL-17 (by 77 and 45% respectively) (Figure 4). The percentages indicate the changes in the two time points tested- day 17 and day 22; post induction. The production of the anti-inflammatory cytokine IL-10 was slightly elevated, but was not statistically significant (Figure 4).

As can be seen in Figure 4E and 4F, FN14•TRAIL reduced the level of ROR-γt by 55 and 30% similar to the reduction in the level of IL-17. In parallel, an increase in the expression of GATA-3, was found, supporting the role of FN14•TRAIL in skewing Th lineage development from Th1 and Th17 to Th2.

### Fn14•TRAIL decreases the number of CCR5-positive T cells in spleens of treated mice

**Figure 5:**
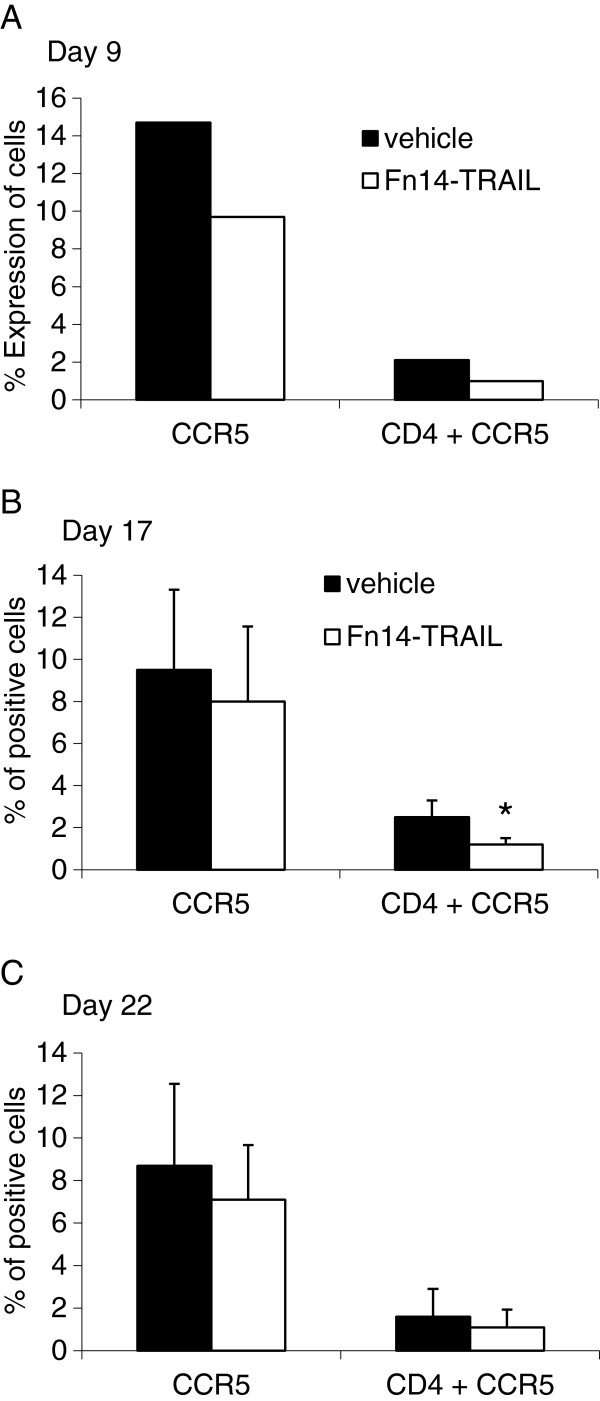
**Fn14·TRAIL alters subpopulations of CCR5+ expressing cells, particularly CCR5+Tcells during different stages of experimental autoimmune encephalomyelitis (EAE). (A)** Splenocytes derived from vehicle and Fn14·TRAIL- treated (200 μg/day) EAE mice, from the day of induction were analyzed for expression of surface markers by fluorescence-activated cell sorting (FACS) analysis as described in Materials and Methods. Expression of CCR5 alone and together with CD4+ for the total splenocytes **i**s shown and is based on the number of cells expressing each marker. **(B –C)** Splenocytes removed on day 17 or day 22 post-induction from mice were treated with 200 μg/day from day 10 post-induction for 7 days, *P <0.05.

We tested the possibility that one of the mechanisms by which Fn14·TRAIL affects inflammation involves alteration of immune cell populations. The phenotype of the splenocytes was characterized by FACS analysis: There was no significant difference in the percentage of CD3, CD4, CD8, CD25, B220, CD19 and CD11b-expressing cells between the placebo-treated and the Fn14·TRAIL-treated mice tested on day 9, 17 and 22 post-induction. Yet, the expression of the murine chemokine receptor CCR5 was reduced (34, 16 and 19%, respectively). The reduction was more pronounced for the CD4+CCR5+ cells (53, 52 and 32%, respectively) (Figure 5A,B,C).

### Abs against Fn14 or TRAIL not detected

As Fn14·TRAIL is composed of human proteins, we tested the possibility that the effect obtained might be due to the antibodies formed against it itself, which could be inhibitory to the naturally occurring murine Fn14 or TRAIL. Using immune-blotting, no antibodies were detected in the serum of treated mice by day 40 when treatment lasted for 16 days. In repeated experiments, we detected a low level of Abs against Fn14·TRAIL (only at 1:20 titer), but not against Fn14 or TRAIL, supporting the notion that Abs against TRAIL or Fn14 are not responsible for the clinical effect of Fn14·TRAIL.

Furthermore, upon testing the safety of Fn14·TRAIL, no apparent toxicity could be detected in livers, lungs, kidneys, gut and hearts of animals treated with up to 200 μg/day These data show that Fn14·TRAIL is not toxic to mice when injected s.c. for 14 days.

### Fn14•TRAIL induces apoptosis in T-cell lines

**Figure 6:**
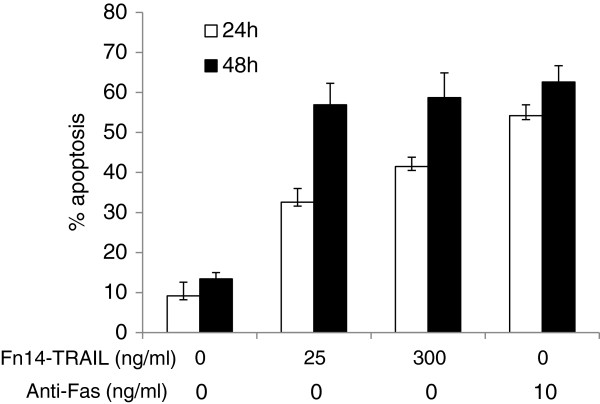
**Effect of Fn14·TRAIL *in vitro *on Jurkat cells.** Jurkat cells were exposed to two concentrations of Fn14·TRAIL for 24 and 48 h, as well as to a/FAS as positive control. Apoptosis was measured using an Annexin-PI kit**,** as described in Materials and Methods. The extent of apoptosis induced by Fn14·TRAIL after 24 and 48 h was 2.5 and 3.5 fold, respectively in the presence of 300 ng/ml Fn14·TRAIL.

We tested whether Fn14·TRAIL is capable of inducing apoptosis in T cells. For this purpose, Jurkat T cell line cells were incubated with Fn14•TRAIL for 24 to 48 h and apoptosis was detected by flow-cytometry. At 24 h 30% of the cells, and at 48 h up to 60% of the cells, were apoptotic (Figure 6), supporting the notion that Fn14•TRAIL induces T cell apoptosis.

When Jurkat cells were cocultured with Fn14·TRAIL and TWEAK, the natural ligand of Fn14, 100% of the cells underwent apoptosis. Tweak itself had no effect on these cells.

It should be noted that the inhibitory effect of Fn14•TRAIL *in vitro* on mice MOG_35-55_ or ConA stimulated splenocytes was evident only when a very high dose of Fn14•TRAIL was used. At concentrations of 3000 ng/ml of Fn14•TRAIL, ConA-induced splenocyte proliferation was inhibited by 24%, and MOG_35-55_ induced proliferation was inhibited by 23%. At concentration of 1500 ng/ml, Fn14•TRAIL’s presence induced only 10% inhibition of ConA-induced splenocyte proliferation, and 25% of the response to MOG_35-55_ stimulation.

## Discussion

In the present study we demonstrate that Fn14·TRAIL protein significantly downregulates EAE. Fn14•TRAIL could affect several stages in the cascade of events leading to CNS inflammation. It is conceivable that it can act already in the early stages of the disease by inhibiting the reactivity of encephalitogenic T cells. When administered by daily injection from day 10 post-disease induction, Fn14·TRAIL significantly attenuated clinical disease severity, as well as the number of CNS inflammatory foci.

MS and EAE are autoimmune inflammatory diseases in which cytokines are extensively involved. Th1 and Th17 cytokines, which play a role in the induction of CNS inflammation and demyelination and in the pathogenesis of EAE and MS [23,24], were markedly reduced by Fn14·TRAIL.

Upon studying the effects of Fn14·TRAIL on specific cell types, we found a decreased number of CCR5+ cells, particularly CD4+CCR5+. CCR5 is expressed mainly on T cells and is associated with a Th1 immune response [25,26].

Fn14·TRAIL affects both Th1 and Th17 cells, the two believed to take an active part in the evolvement of EAE. This was the case when Fn14·TRAIL was given during the priming phase after immunization and splenocytes were harvested on day 9 post-immunization and also when Fn14·TRAIL was given from day 10 for 7 days on day 17 or 22 post-induction. This is in agreement with previous work describing the inhibition of EAE by *in vivo* expression of Fn14•TRAIL [20]. In our study, however, we treated the animals for a much shorter time, with a very significant inhibitory effect.

Fn14·TRAIL has two potentially active domains. The first, Fn14, is the natural receptor for TWEAK, which has been described as playing an enhancing role in EAE [13,18,27]. Blocking Fn14: TWEAK has been suggested to have a beneficial effect in the treatment of inflammatory diseases [28,29], EAE specifically [19]. This suggestion is based on the ability of TWEAK to induce the secretion of pro-inflammatory cytokines such as IL-6 and MCP-1 [30], but mainly on the finding that blocking of the TWEAK:Fn14 interaction reduced disease severity in EAE [19,27]. We observed that Fn14·TRAIL lowered disease severity only when given from day 10 after MOG immunization. Treatment of the mice from day 1 did not yield the same effect. This is in agreement with the previous findings of Desplat-Jego *et al*. who showed that blocking anti-TWEAK Abs given from day 9 after MOG immunization significantly inhibited clinical symptoms of EAE in mice, and decreased the number of CNS inflammatory foci [27]. Of note, anti-TWEAK Abs did not affect the *ex vivo* T cell response to MOG_35-55_ re-challenge when given on day 9 from immunization. Fn14·TRAIL did inhibit the T-cell response to *ex vivo* restimulation, highlighting the difference between the two agents and suggesting that more than one mechanism is involved in Fn14·TRAIL’s inhibitory effect. Importantly, although Fn14·TRAIL has a short half-life compared with that of antibodies, its effect was as long lasting as that of the Abs administered. This might be due to its influence on pathogenic T cell migration to the CNS. Such an effect has been suggested also by Desplat-Jego *et al*. [18,27] and by Razmara *et al*. [20].

The other domain of Fn14·TRAIL is the extracellular moiety of TRAIL. Previous studies support the importance of TRAIL in the amelioration of EAE severity. Soluble TRAIL has been shown to suppress the disease in mice [31] and blocking of TRAIL, especially when given just before the first clinical signs of the disease appeared, resulted in significant augmentation of symptoms. [6,31]. Moreover, when EAE was induced by MOG-expressing dendritic cells (DCs) disease severity was hampered if these DCs also expressed TRAIL [7]. Interestingly, other studies, using adoptive transfer of TRAIL ^−/−^ lymphocytes, have shown that TRAIL has a neurodegenerative effect on CNS-infiltrating lymphocytes inducing demyelination, and that selective blocking of TRAIL’s activity by intracisternal administration of DR5:Fc fusion protein, successfully inhibited the clinical and pathological signs of EAE [32]. Intriguingly, Fn14•TRAIL can be anchored to TWEAK- expressing DCs and present TRAIL to TRAIL-receptor bearing cells, such as activated T cells, thus mimicking the genetically engineered TRAIL-expressing DCs. Also, as it has been shown to interfere with BBB permeability, these DCs will act mainly in the periphery and not in the CNS [20].

The ability of Fn14•TRAIL to inhibit murine splenocytes’ response to stimulation, either antigen specific or mitogenic, could be explained by its ability to induce apoptosis through the TRAIL moiety or to inhibit proliferation (by interfering with the TWEAK: Fn14 axis). The finding that Fn14•TRAIL is capable of inducing apoptosis in the Jurkat T cell line supports the first assumption.

An interesting finding from our experiments is that Fn14·TRAIL inhibited clinical signs of disease only when given closer to time of disease onset, and had no significant effect when given from time of immunization, as was described for blocking TWEAK or TRAIL [27]. There are several possible explanations for this phenomenon. It may be that early treatment affects different cell populations, changing the balance between effector and regulatory cells in a way that facilitates the appearance of clinical disease, and that late treatment affects the effector cells, while partially sparing the regulatory/inhibitory cells. We did not find any difference in the prevalence of total CD4, CD8 or CD25 positive cells in the spleen, but did find a significant decrease in CCR5-expressing CD4 cells. CCR5 has been shown to be essential for T-cell recruitment to the inflammatory site in EAE, and specific targeting of CCR5-expressing cells was shown to be helpful in treating EAE mice [33,34]. Another possibility could be the development of blocking Abs against the human fusion protein given to the mice, resulting in the prevention of its inhibitory effect from day 10 and on. Although we did not detect anti-TRAIL or anti-Fn14 Abs in the sera of treated mice, this possibility cannot be excluded.

## Conclusions

In summary, Fn14·TRAIL proved to be very effective in inhibiting the clinical course of EAE, even when given for a short time from day 10 of induction, and is correlated to the diminished inflammatory response detected in the CNS. Importantly, no signs of toxicity were found in the treated mice. Fn14•TRAIL’s action involves inhibition of the T cell response, particularly of Th1 and Th17, and a decreased number of CCR5^+^CD4^+^ cells. Cumulatively, our findings support the immunomodulatory potential of FN14•TRAIL in the treatment of MS and other autoimmune diseases.

## Abbreviations

BBB: blood–brain barrier; CFA: complete Freund adjuvant; CMV: cytomegalovirus; CNS: central nervous system; EAE: experimental autoimmune encephalomyelitis; DCs: Dendritic cells; ES-DC: embryonic stem cell-derived dendritic cells; FCS: fetal calf serum; FACS: fluorescence-activated cell sorting; FITC: fluorescein isothiocyanate; Fn14: fibroblast growth factor-inducible-14; HPRT: hypoxantine phosphoribosyltransferase; IFN: interferon; I.P: intraperitoneally; LN: lymph node; MOG: myelin oligodendrocyte glycoprotein; MS: multiple sclerosis; S.C.: subcutaneously; PCR: polymerase chain reaction; PI: propidium iodide; TRAIL: tumor necrosis factor-related apoptosis-inducing ligand; TWEAK: TNF-related weak inducer of apoptosis.

## Competing interests

MDE has research grant and consulting contract with KAHR MEDICAL LTD. Other authors declare no competing interests.

## Authors’ contributions

HPH, TM and MITS performed the animal and cell cultures studies. TBP and AA were involved in apoptosis and antibodies detection experiments. TB and MDE were involved in study design, data analysis and manuscript writing. All authors read and approved the final manuscript.
